# Serum heat shock protein 27 levels predict cardiac mortality in hemodialysis patients

**DOI:** 10.1186/s12882-018-1157-1

**Published:** 2018-12-17

**Authors:** Andrzej Jaroszyński, Anna Jaroszyńska, Tomasz Zaborowski, Anna Drelich-Zbroja, Tomasz Zapolski, Wojciech Dąbrowski

**Affiliations:** 1 Institute of Medical Sciences, Jan Kochanowski University in Kielce, Al. IX Wieków Kielc 19A, 25-317 Kielce, Poland; 2 Department of Family Medicine, Medical University of Lublin, Lublin, Poland; 3 Department of Interventional Radiology and Neuroradiology, Medical University of Lublin, Lublin, Poland; 4 Department of Cardiology, Medical University of Lublin, Lublin, Poland; 5 Department of Anesthesiology and Intensive Care, Medical University of Lublin, Lublin, Poland

**Keywords:** Heat shock protein, End-stage renal disease, Mortality, Atherosclerosis, Oxidative stress, Mortality

## Abstract

**Background:**

Decreased heat shock protein 27 (HSP27) participates in many processes that are involved in cardiovascular (CV) disease. The objective of the study was to evaluate if HSP27 level was predictive of mortality as well as to evaluate factors associated with HSP27 level in a group of patients treated with HD.

**Methods:**

Enrolled to the study were 202 HD patients. Clinical data, biochemical, echocardiographic, and carotid atherosclerosis parameters were evaluated. Patients were splited into groups on the basis of the cut-off lower and higher 50th percentile of serum HSP27 levels, and were followed-up for 28.68 ± 6.12 months.

**Results:**

No significant difference was observed between serum HSP27 levels in patients and controls. Low HSP27 patients were older, had higher left ventricular mass index, lower ejection fraction, higher prevalence of diabetes, myocardial infarction and carotid atherosclerosis, higher C-reactive protein level, and worse oxidant/antioxidant status. The multiple regression analysis identified that HSP27 levels were independently, negatively associated with serum oxidized LDL and the number of carotid plaques. Using the Kaplan–Meier analysis it was shown that the cumulative incidences of both CV and sudden cardiac death (SCD) mortality were higher in low HSP27 group in comparison with high serum HSP27 group. A multivariate Cox analysis showed that HSP27 level is an independent and strong predictor of CV as well as SCD mortality.

**Conclusions:**

Low serum HSP27 level is independently associated with both CV and SCD mortality but not with all-cause mortality. Low serum HSP27 level is associated with carotid atherosclerosis and oxidative stress.

## Background

In recent years mortality rate is falling 2–3% per year in hemodialysis (HD) patients, however it is still substantially higher compared with general population, even after controlling for age, gender, and co-morbidities [1, 2]. Cardiovascular (CV) diseases have been identified as the main cause of death in HD patients [1–6]. In particular, sudden cardiac death (SCD) significantly contributes to increased mortality, and is considered the most common cause of death in HD patients [6–9].

Heat-shock proteins (HSPs), initially recognized in *Drosophila melanogaster* in response to heat shock, are present in all cells of all organisms from bacteria to humans [10–13]. HSPs are highly conserved chaperone proteins that interact with other proteins to facilitate normal cellular functions. HSPs play crucial roles in folding and unfolding of proteins, assembly of multiprotein complexes, transport and sorting of proteins into correct subcellular compartments, cell-cycle control and signaling, and protection of cells against different forms of cellular stress, including oxidative stress, as well as apoptosis [11–17]. Heat shock protein 27 (HSP27) belongs to the small molecular weight HSP family, and appears to serve a significant role in CV system. HSP27 is considered both a potential biomarker of disease and injury as well as potential therapeutic target [18, 19]. It has been demonstrated that HSP27 functions as an antioxidant, exerts cytoprotective effects, inhibits apoptosis, attenuates medication- as well as endotoxin-induced myocardial injury [11–13, 15, 16, 20, 21]. Very recent studies revealed that in humans low serum HSP27 levels predict atrial fibrillation recurrence after catheter ablation [20] and are inversely associated with plaque burden in coronary artery and prognosis of future adverse clinical events [19].

Given the role of HSP27 plays in CV system and that chronic inflammation, oxidative stress, and enhanced apoptosis are characteristic features of renal failure we thought that HSP27 could be a potential biochemical indicator useful in risk stratification in patients treated with HD.

The objective of the study was to: (1) prospectively evaluate if HSP27 was predictive of all-cause, CV as well as SCD mortality in a group of patients treated with HD, and (2) to assess the possibile associations between HSP27 and biochemical, echocardiographical as well as clinical factors.

## Material and methods

### Patients

Included to the study were adult patients treated with HD at two dialysis units in Lublin (Poland) from March 2010 to May 2015. The following exclusion criteria were applied: HD treatment less than 3 month (to include exclusively patients with end-stage kidney disease), advanced neoplastic diseases reducing the chance of survival for 3 months, and patients displaying symptoms of acute infections at baseline (to reduce the possible influence of transient factors on HSP27 level). Given that it was not possible to estimate population size meeting the criteria used in our study, the sample size calculation was not performed. Included were all available HD patients in Lublin. Informed consent was obtained from all participating patients and the studies were approved by the Ethical Committee of Medical University of Lublin.

### Biochemical variables

Routine tests including electrolytes, hemoglobin, creatinine, urea, C-reactive protein (CRP), total protein, albumin, intact parathormone (PTH), lipids profile, and troponin T were measured by automated analyzers. Serum total antioxidant status (TAS) as well as serum total oxidant status (TOS) were evaluated by using colorimetric method (Immundiagnostik AG, Germany). TAS assay uses the ability of antioxidants in the serum to inhibit the formation of ABTS^+^ (2,2′-Azino-di-[3-ethylbenzthiazoline sulphonate]) from the oxidation of ABTS by metmyoglobin (a peroxidase). TOS assay is based on the oxidation of ferrous ion to ferric ion in the presence of various oxidative species. Serum HSP27, NT-proBNP, and oxidized LDL (oxLDL) were measured by the ELISA method (Biomedica). All measurements were taken at the beginning of the evaluation, the day after the dialysis session.

### Control group

Serum HSP27 was evaluated in 42 gender- (21 F and 21 M) and age-matched (69.2 ± 6.1 years) controls. The control group consisted of healthy volunteers, having no abnormalities detected by physical examination, chest X-ray and laboratory analysis.,

### Echocardiographic examination

All echocardiographic measurements were performed by an experienced cardiologist who was blinded to the clinical data of the study subjects in the morning after dialysis [22] accordingly to the American Society of Echocardiography recommendations [22, 23]. LVH was diagnosed when LVMI exceeded 130 g/m^2^ in males or exceeded 110 g/m^2^ in females.

### Evaluation of carotid atherosclerosis

Examination of the carotid arteries was performed in a B mode presentation using the ultrasound system GE LOGIQ 500 with a 6–12 MHz linear transducer as described in detail previously [24]. Intima-media thickness (IMT) as well as the number of plaques were measured in all patients.

### Follow-up data

From the day of baseline assessment patients were followed for 36 months or until the date of death or kidney transplantation. The end points of the study were all-cause mortality, CV mortality and SCD. The definition of CV death was in line with that presented in Standardized Definitions for End Point Events in Cardiovascular Trials [25]. SCD was defined according to Hemodialysis (HEMO) trial [6].

### Statistical analysis

Statistical analysis was carried out using Statistica Version 10 as described in detail previously [26]. Linear regression analysis was performed by using the Pearson or Spearman test, as appropriate. When non-normally distributed, data were transformed logarithmically before analysis was carried out. For further analysis, patients were splited into groups using the cut-off lower and higher 50th percentile of serum HSP27 levels (low HSP27 and high HSP27 groups, respectively). Significance of differences between low and high HSP27 groups was assessed using *t*-Students test. Multiple stepwise regression analysis was performed to estimate the potential influence of various factors on HSP27 level. Qualified to the model were parameters that displayed differences with a *p* value < 0.05 between high and low HSP27 groups. The prognosis value of serum HSP27 for predicting the study outcomes was assessed by the Kaplan–Meier method (the log-rank test) for all-cause mortality, CV mortality as well as SCD. Cox proportional hazard regression analysis was used to analyze relations between baseline parameters and endpoints. In multivariate Cox models, all variables that reached the value of *p* < 0.15 in the univariate analysis were included. A statistical significance level of *p* < 0.05 was used.

## Results

Enrolled to the study were 202 HD patients (107 females and 95 males), aged 41–91 years (mean 70.1 ± 8.13), who were treated by HD from 1 to 167 months (mean 47.54 ± 26.72). Patients had the following causes of kidney failure: diabetes mellitus (*n* = 84), glomerulonephritis (*n* = 41), hypertensive nephropathy (*n* = 21), polycystic kidney disease (n = 8), obstructive nephropathy (*n* = 6), chronic pyelonephritis (*n* = 5), and unknown/unsure (*n* = 37). Out of 202 patients who qualified to the study, 80.2% were treated with angiotensin-converting enzyme inhibitors or angiotensin receptor blockers, 87.1% were taking beta-blockers, and 63.4% received statin. Previous myocardial infarction (MI) was diagnosed in 28.2% of patients, hypertension was present in 83.7% of patients, 54.0% of patients were diabetic. Systolic dysfunction was diagnosed in 25.2% of patients, whereas LVH occured in 64.9% of enrolled patients.

Serum HSP27 levels did not differ between patients and controls (30.13 ± 9.09 μg/l and 34.06 ± 8.16 μg/l, *p* = 0.101). No difference was found between HSP27 levels in HD females and males (29.89 ± 8.44 vs. 30.34 ± 8.75, *p* = 0.673).

Low and high HSP27 groups were created on the basis of the cut-off lower and higher 50th percentile of serum HSP27 levels. Patients qualified to the low HSP27 group were older (*p* = 0.009), had higher prevalence of MI (*p* < 0.001), as well as diabetes (*p* = 0.007). With regard to echocardiographic parameters, low HSP27 patients had higher LVMI (*p* < 0.001), and lower EF (p < 0.001) compared to individuals with high HSP27 levels. In the case of biochemical indices, low HSP27 patients had higher CRP (*p* = 0.006) TOC (p < 0.001) as well as oxLDL levels (*p* < 0.001), whereas TAC level was lower (*p* = 0.011). Low HSP27 subjects had also higher IMT value (*p* = 0.008) and increased plaque burden (*p* < 0.001). Basic demographic data, clinical and biochemical data of HD patients are presented in Table 1.

**Table 1:** Basic demographic data, clinical and biochemical data of patients

parameter	All patients *n* = 202	Low HSP27 *n* = 101	High HSP27 n = 101	p
Age (years)	70.1 ± 8.13	71.8 ± 7.78	68.4 ± 8.21	0.009
HD vintage (months)	57.54 ± 26.72	58.11 ± 25.34	57.02 ± 26.16	0.387
MI (%)	28.2	34.7	21.8	< 0.001
Diabetesmellitus (%)	54.0	57.4	50.5	0.007
Hypertension (%)	83.7	87.1	80.2	0.187
Smoking	18.3	16.8	19.8	0.223
Beta-blockers (%)	87.1	85.1	89.1	0.278
ACE/ARB (%)	80.2	79.2	82.2	0.301
Statins (%)	63.4	64.4	62.4	0.693
LVMI (g/m^2^)	144.2 ± 42.74	159.2 ± 38.68	129.2 ± 41.83	< 0.001
EF (%)	56.83 ± 6.33	53.72 ± 6.21	60.02 ± 5.96	< 0.001
Hemoglobin (g/dL)	11.27 ± 1.11	11.63 ± 1.08	10.90 ± 1.02	0.098
Total cholesterol (mg/dL)	187.4 ± 37.35	188.1 ± 37.11	186.0 ± 35.87	0.674
LDL cholesterol (mg/dL)	116.9 ± 31.03	116.1 ± 30.13	118.3 ± 28.02	0.632
HDL cholesterol (mg/dL)	43.12 ± 18.11	43.4 ± 17.86	42.67 ± 14.45	0.711
Triglycerides (mg/dL)	172.1 ± 61.24	170.2 ± 59.54	175.7 ± 54.9	0.314
PTH, range (pg/mL)	384 (0.0–1212)	355 (0.0–924)	448 (0.0–1212)	0.217
Albumin (g/dL)	3.68 ± 0.37	3.69 ± 0.36	3.68 ± 0.32	0.825
CRP, range (mg/dL)	7.28 (0.19–112.1)	12.72 (0.019–112.1)	5.04 (0.22–47.4)	0.006
Troponin T, range (μg/L)	0.059 (0.00–0.773)	0.071 (0.00–0.773)	0.044 (0.032–0.775)	0.084
*NT-proBNP (f*mol/ml)	314.3 ± 105.4	338.7 ± 105.5	295.9 ± 111.8	0.188
Sodium (mmol/L)	137.8 ± 2.63	137.7 ± 2.61	138.0 ± 2.69	0.622
Potassium (mmol/L)	5.70 ± 0.65	5.72 ± 0.67	5.69 ± 0.64	0.742
Calcium (mmol/L)	2.47 ± 0.23	2.46 ± 0.22	2.48 ± 0.24	0.513
Phosphate (mmol/L)	2.24 ± 0.37	2.18 ± 0.21	2.30 ± 0.23	0.103
Ca x P product mg^2^/dl^2^	48.44 ± 9.68	46.99 ± 9.28	49.72 ± 9.34	0.211
TAC (μmol/l)	250.4 ± 31.90	238 ± 31.32	261 ± 32.63	0.011
TOC (μmol/l)	358 (48–1275)	611 (197–1275)	277 (48–623)	< 0.001
oxLDL (mg/L)	1.56 ± 0.43	1.83 ± 0.42	1.39 ± 0.41	< 0.001
IMT (mm)	0.845 ± 0.276	0.886 ± 0.268	0.793 ± 0.274	0.008
Number of plaques (n)	3.86 ± 2.13	4.62 ± 1.63	3.21 ± 1.77	< 0.001

*CAD* coronary artery disease, *MI* history of myocardial infarction, *ACE/ARB* angiotensin-converting enzyme inhibitors/angiotensin receptor blockers, *LVMI* Left ventricular mass index, *LVH* Left ventricular hypertrophy, *EF* Ejection fraction, *PTH* parathormon, *CRP* C-reactive protein, *NT-proBNP* N-terminal pro-hormone brain natriuretic peptide, *TAC* total antioxidant capacity, *TOC* total oxidant capacity, *oxLDL* oxidized LDL, *IMT* intima-media thickness

The results of multiple regression analysis showed that HSP27 levels were independently inversely associated with: (i) oxLDL and (ii) number of atherosclerotic plaques (Table 2).

**Table 2:** Factors influencing HSP27 estimated by multivariate stepwise regression analysis

Dependent variable	Independent variables	B	St. error	Beta	P
HSP27	oxLDL	- 0.502	0.027	0.351	0.001
	Number of plaques	- 12.23	5.61	0.289	0.012
	Model (*R* = 0.652, R^2^ = 0.396)				

The average follow-up period was 28.68 ± 6.12 months (range 2–36 months). During the follow-up period 71 death for any reason were reported (35.1%), and mortality rate was 11.7% per year. Deaths from CV causes accounted for 49.3% of all death, and SCD accounted for 25.3% of all deaths. Malignancy and infectious complications were responsible for 9.9 and 12.6% of all death, respectively. All other reasons as well as unknown and unsure reasons were responsible for 28.2% of all death. Renal transplantation was performed in 12 patients.

**Fig. 1:**
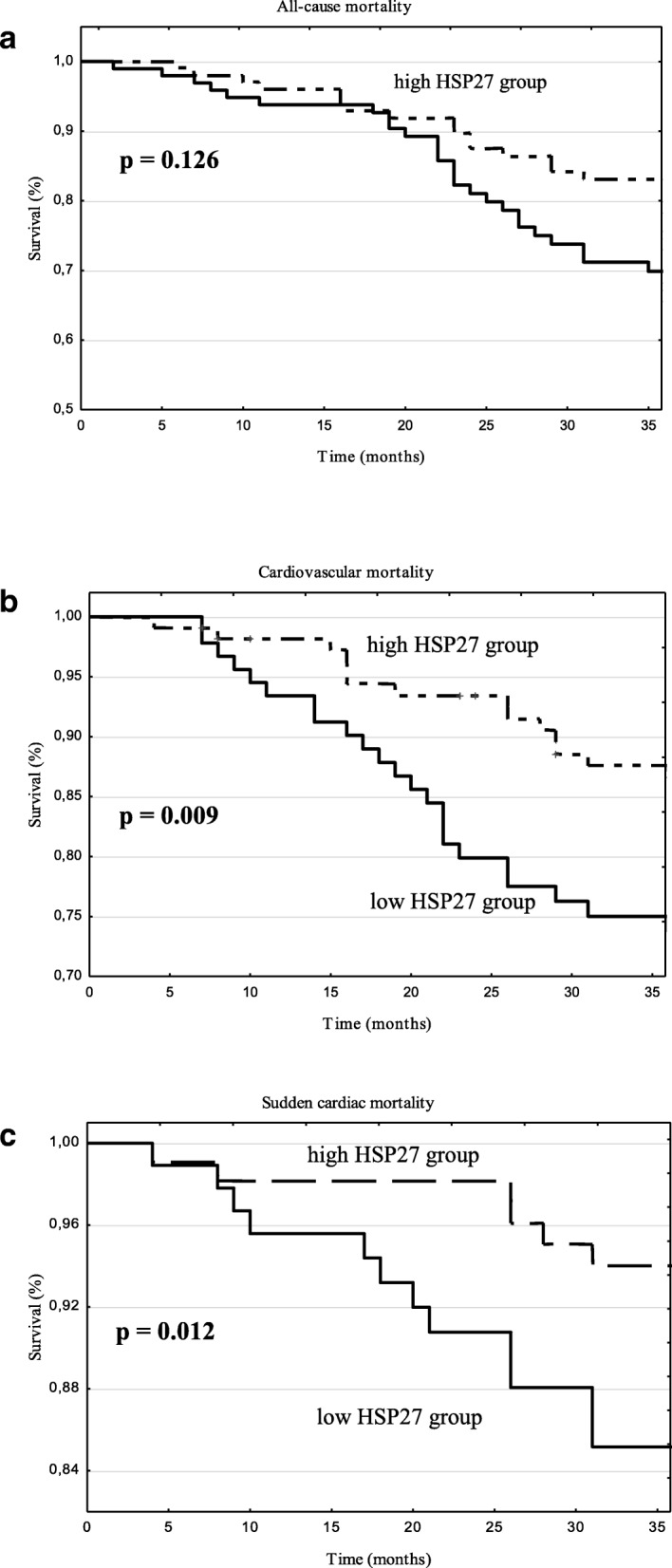
Kaplan–Meier survival plots for (**a**) all-cause mortality, (**b**) cardiovascular mortality as well as (**c**) sudden cardiac mortality in hemodialysis patients stratified by 50th percentile of serum HSP27 level

Using the Kaplan–Meier analysis it was shown that the cumulative incidence of death for any reason did not differ between low and high HSP27 groups (log-rank, *p* = 0.126; Fig. 1a). It was also revealed that the cumulative incidence of both CV and SCD mortality was higher in low HSP27 patients compared to high HSP27 patients (log-rank, *p* = 0.009 and *p* = 0.005, respectively; Fig. 1b and c, respectively). In order to control for possible confounders Multivariate Cox analysis was performed. Entered to the model were univariate predictors of cardiac mortality. The results of both univariate and multivariate Cox analyses are presented in Tables 3, 4, 5. In the case of all-cause mortality the independent predictors were age [hazard ratio (HR) 1.97, *p* < 0.001], hemoglobin [HR 1.34, *p* = 0.006], and CRP [HR 1.38 *p* = 0.012]. In the case of CV mortality the independent predictors were age [HR 1.68, p < 0.001], HSP27 [HR 3.23, p < 0.001], and troponin T [HR 1.72, *p* = 0.008]. In the case of SCD the independent predictors were age [HR 1.30, p < 0.001], HSP27 [HR 2.75, *p* = 0.001], and EF [HR 1.31, p = 0.008].

**Table 3:** Uni- and multivariate predictors of all-cause mortality

Parameter	Univariate HR (95% CI)	p	Multivariate HR (95% CI)	p
Age	2.48 (1.93–2.81)	< 0.001	1.97 (1.48–2.31)	< 0.001
Diabetes mellitus	1.47 (0.85–1.99)	0.008	1.22 (0.57–1.85)	0.327
History of MI	1.41 (0.91–1.75)	0.007	1.48 (0.73–2.45)	0.181
LVMI	1.43 (0.62–2.45)	0.224		
EF	2.321 (1.75–2.97)	0.092	1.93 (0.68–3.21)	0.367
TOC	1.84 (1.03–2.46)	0.186		
TAC	1.56 (0.91–3.43)	0.314		
oxLDL	1.79 (0.83–3.15)	0.169		
Phosphate	2.68 (1.56–4.18)	0.325		
Hemoglobin	1.67 (1.27–2.35)	0.003	1.34 (0.87–1.81)	0.006
Troponin T	1.57 (0.67–2.38)	0.186		
CRP	1.72 (1.27–2.19)	0.002	1.38 (0.76–1.93)	0.012
IMT	1.02 (0.36–1.75)	0.376		
Number of plaques	0.93 (0.34–1.89)	0.385		
HSP-27	2.64 (1.89–3.46)	0.009	2.19 (1.18–3.81)	0.106

In multivariate analysis included were all variables with a p value < 0.15 in the univariate analysis. *HR* hazard ratio, *CI* confidence interval, *MI* myocardial infarction, *LVMI* left ventricular mass index, *EF* ejection fraction, *TOC* total oxidant capacity, *TAC* total antioxidant capacity, *oxLDL* oxidized LDL, *CRP* C-reactive protein, *IMT* intima-media thickness, *HSP-27* heat shock protein 27. In the multivariate analyses, parameters with a *p* ≤ 0.15 were entered

**Table 4:** Uni- and multivariate predictors of cardiovascular mortality

Parameter	Univariate HR (95% CI)	p	Multivariate HR (95% CI)	p
Age	2.18 (1.51–2.71)	< 0.001	1.68 (1.25–2.21)	< 0.001
Diabetes mellitus	1.13 (0.49–2.27)	0.274		
History of MI	1.51 (0.92–2.24)	0.008	1.39 (0.71–2.49)	0.136
LVMI	2.41 (1.65–3.23)	0.105	1.86 (0.93–3.12)	0.345
EF	1.28 (0.61–2.45)	0.219		
TOC	1.75 (1.26–2.44)	0.011	1.53 (1.11–2.79)	0.214
TAC	1.86 (1.01–3.44)	0.257		
oxLDL	1.49 (0.93–2.65)	0.163		
Phosphate	0.89 (0.33–1.97)	0.311		
Hemoglobin	1.62 (0.98–2.67)	0.247		
Troponin T	1.84 (1.35–2.59)	0.007	1.72 (1.24–2.34)	0.008
CRP	1.35 (0.75–1.82)	0.024	1.15 (0.43–1.98)	0.211
IMT	1.46 (0.85–1.83)	0.019	1.37 (0.52–2.11)	0.136
Number of plaques	1.23 (0.63–2.29)	0.279		
HSP-27	3.97 (3.49–4.58)	< 0.001	3.23 (2.67–3.71)	< 0.001

In multivariate analysis included were all variables with a p value < 0.15 in the univariate analysis. *HR* hazard ratio, *CI* confidence interval, *MI* myocardial infarction, *LVMI* left ventricular mass index, *EF* ejection fractionm, *TOC* total oxidant capacity, *TAC* total antioxidant capacity, *oxLDL* oxidized LDL, *CRP* C-reactive protein, *IMT* intima-media thickness, *HSP-27* heat shock protein 27. In the multivariate analyses, parameters with a p ≤ 0.15 were entered

**Table 5:** Uni- and multivariate predictors of sudden death mortality

Parameter	Univariate HR (95% CI)	p	Multivariate HR (95% CI)	p
Age	1.68 (1.12–2.71)	< 0.001	1.30 (0.95–1.76)	< 0.001
Diabetes mellitus	1.54 (0.52–2.67)	0.356		
History of MI	1.72 (0.65–2.68)	0.198		
LVMI	2.68 (1.87–3.83)	0.285		
EF	1.47 (0.83–1.94)	0.002	1.31 (0.79–1.74)	0.008
TOC	2.15 (1.12–3.94)	0.239		
TAC	1.37 (0.62–2.56)	0.311		
oxLDL	1.71 (0.101–2.98)	0.072	1.46 (0.77–3.18)	0.205
Phosphate	1.19 (0.41–1.99)	0.275		
Hemoglobin	1.32 (0.68–2.07)	0.235		
Troponin T	1.94 (1.35–2.48)	0.011	1.43 (0.66–2.24)	0.113
CRP	0.95 (0.55–1.62)	0.278		
IMT	1.71 (0.41–2.87)	0.312		
Number of plaques	1.41 (0.53–2.69)	0.244		
HSP-27	3.18 (2.68–3.97)	< 0.001	2.75 (2.37–3.44)	0.001

In multivariate analysis included were all variables with a p value < 0.15 in the univariate analysis. *HR* hazard ratio; *CI* confidence interval, *MI* myocardial infarction, *LVMI* left ventricular mass index, *EF* ejection fraction, *TOC* total oxidant capacity, *TAC* total antioxidant capacity, *oxLDL* oxidized LDL, *CRP* C-reactive protein, *IMT* intima-media thickness, *HSP-27* heat shock protein 27. In the multivariate analyses, parameters with a p ≤ 0.15 were entered

## Discussion

Three key findings were generated in our study: (1) serum HSP27 did not differ between patients compared with controls, (2) low serum HSP27 is associated with carotid atherosclerosis and oxidant status, (3) low serum HSP27 level is an independent predictor of CV mortality as well as SCD but not all-cause mortality in HD patients.

To our knowledge this is the first study to compare serum HSP27 levels between HD patients and controls. The lack of difference in serum HSP27 between HD patients and controls is suprising. It may be, however, the resultant of two processes: renal function and the accumulation of CV diseases. Data on the effect of renal function on HSP27 levels are divergent. Some authors have demonstrated a significant association between HSP27 and renal function both in healthy volunteers [27] and in non-dialysis, non-diabetic chronic kidney disease patients [28]. Others, however, have found that HSP27 plasma levels were not influenced by kidney function [29]. Similarly, data on the effect of various CV diseases on HSP27 levels are divergent. Traxler et al. [29] have found elevated HSP27 serum concentrations in patients with heart failure. Other studies have documented, however, an inverse relation between HSP27 plasma levels and multiple forms of atherosclerosis, such as coronary artery disease, abdominal aortic aneurysm and peripheral artery disease [13, 19, 30, 31]. In HD patients both heart failure and atherosclerosis are prevalent. Thus, the relationship of HSP27 with renal function and its pathophysiology remain to be evaluated in further studies.

Moreover, results of the present study have revealed negative association between carotid atherosclerosis expressed as the number of plaques and serum HSP27 levels. Given that carotid artery atherosclerosis mirror either general atherosclerosis or atherosclerotic changes located in coronary artery, our results are consistent with previous studies that found a strong inverse relationship between serum HSP27 levels and plaque burden in coronary arteries [19] as well as carotid atherosclerosis [30]. It has been also reported in a small cohort of HD patients that serum HSP27 inversely correlated with carotid IMT [32]. Our results are also consistent with experimental studies that documented the inverse relationship between HSP27 and atherosclerosis [11, 18, 31, 33], and that increasing serum HSP27 levels attenuated the develompent of atherosclerosis and shifted palques to a more stable morphology [19].

Our study has revealed that decreased HSP27 level was independently associated with oxidative stress, expressed as increased oxLDL level. These results are in agreement with previous studies which revealed that HSP27 functions as antioxidant [11–14, 16, 17, 34]. Strong evidence suggests that HD patients experience imbalance between oxygen reactive species (ROS) production and antioxidant defenses leading to enchanced state of oxidative stress. It has been hypothesized that oxidative stress and its sequel strongly contribute to increased CV mortality in HD patients [35–41]. ROS modifies LDL particles, turing them into oxLDL. Oxidatively modified LDL particles exhibit proinflammatory and proatherogenic effects in vessel walls [41–43]. HSP27 functions as antioxidant lowering the levels of ROS both by decreasing intracellular iron and increasing intracellular levels of glutatione [14]. HSP27 may affects oxLDL’s contribution to atherogenesis both by competing with oxLDL uptake into macrophages and by lowering ROS production in endothelial cells, diminishing oxidative modification of LDL [18, 43]. Additionally, in low HSP27 group higher TOC and lower TAC levels were observed.To the best of our knowledge, this is the first study that showed the link between HSP27 and both TOC and TAC. The results of our study, however, did not found both TOC and TAC as independent predictors of mortality. Our results are contrary to the results of Antunovic et al. [44], who found that TAS was an independent predictor of the all-cause mortality in a group of HD patinets. Given the above, HSP27 seems to be a molecule linking cardiovascular mortality with oxidative stress in HD patients.

In this study we demonstrated for the first time that plasma levels of HSP27 are a potential biomarker for predicting both CV and SCD mortality in HD patients. A significant separation of the Kaplan–Meier curves was observed early after the beginning of the follow-up and and it remained separated until the end of follow-up for both CV and SCD mortality. Additionally, after controlling for possibile confounders multivariate Cox analysis showed that HSP27 remained an independent predictor of both CV and SCD mortality. Considering the high prevalence of CV diseases and high moratlity rate of cardiac events, early identification of patients at risk of increased CV mortality, including SCD is of clinical significance. The prognostic value of HSP27 for estimating the risk of CV mortality in HD patients is in line with previous study of Seibert et al. [19], who revealed that low HSP27 was predictive of CV events in patients with coronary artery disease. HSP27 may exhibit cardioprotective effects by its antioxidant and anti-apoptotic properties, the role in attenuating atherogenesis by modifying lipid uptake and inflammation in the plaque as well as the ability of HSP27 to protect the endothelium from ischemia [11, 12, 14, 18, 33, 43]. A particularly interesting issue, although impossible to clarify at the present stage of our study, is the question whether HSP27 is only an indicator of poor prognosis or whether it potentially identifies pathogenic mechanisms underlying the increased CV as well as SCD mortality. In our study serum HSP27 levels were not predictive of all-cause mortality. HSP27 plays a significant role in carcinogenesis, and may have potential clinical uses as biomarker for cancer diagnosis, for assessing disease progression, or as therapeutic targets for cancer therapy [45]. Given that malignancy is prevalent in HD patients lack of association between HSP27 and all-cause mortality may result from the dichotomous role of HSP27 in CV disease versus cancer.

The limitations of the study include relatively small number of subjects, however, it was sufficient to show a predictive value for HSP27 concentration. Our results are merely descriptive and a pathophysiologic explanation for the impact of HSP27 needs to be part of further studies. Finally, it is likely that serial instead of single measurements of HSP27 levels may influence the results, which will make HSP27 a more or less useful parameter in determining clinical events in HD patients.

## Conclusions

Low serum HSP27 concentration is independently associated with CV as well as SCD mortality but not with all-cause mortality. Low serum HSP27 level is associated with carotid atherosclerosis and oxidative stress.

## Abbreviations

ACE/ARB: Angiotensin-converting enzyme inhibitors/angiotensin receptor blockers
CAD: Coronary artery disease
CRP: C-reactive protein
CV: Cardiovascular
ESRD: End-stage renal disease
HD: Hemodialysis
HSP: Heat-shock protein
IMT: Intima-media thickness
LVH: Left ventricular hypertrophy
LVMI: Left ventricular mass index
MI: Myocardial infarction
NT-proBNP: N-terminal pro-hormone brain natriuretic peptide
oxLDL: Oxidized LDL cholesterol
PTH: Intact parathormone
SCD: Sudden cardiac death
TAS: Total antioxidant status
TOS: Total oxidant status
